# Telemedicine and its transformation of emergency care: a case study of one of the largest US integrated healthcare delivery systems

**DOI:** 10.1186/s12245-017-0146-7

**Published:** 2017-07-06

**Authors:** Rahul Sharma, Peter Fleischut, Daniel Barchi

**Affiliations:** 1 Department of Emergency Medicine, New York Presbyterian-Weill Cornell Medicine, 525 E 68St. M-130, New York, NY 10021 USA; 2 Department of Information Technology, New York Presbyterian Hospital, New York, NY USA; 3 Department of Anesthesiology, Weill Cornell Medicine, New York, NY USA

**Keywords:** Telemedicine, Telehealth, Emergency Medicine, Stroke, Digital health

## Abstract

Innovative methods for delivering healthcare via the use of technology are rapidly growing. Despite the passage of the Affordable Care Act, emergency department visits have continued to rise nationally. Healthcare systems must devise solutions to face these increasing volumes and also deliver high quality care. In response to the changing healthcare landscape, New York Presbyterian Hospital has implemented a comprehensive enterprise wide digital health portfolio which includes the first mobile stroke treatment unit on the east coast and the first emergency department-based digital emergency care program in New York City.

## Background

US healthcare systems have recently shown significant interest in the digital arena, specifically telemedicine. The use of telemedicine has grown rapidly and is now employed in all aspects of healthcare, in outpatient care, inpatient care, nursing home care, workplace, and consumer homes. The American Telemedicine Association reports there are currently 200 active telemedicine networks with over half of all US hospitals now using some form of telemedicine [1]. Over the past few decades, the healthcare industry has increasingly engaged and, to some extent, driven technological advancement and innovation. As a result, telemedicine has metamorphosed into what it is today. Today’s physician works with high-resolution video, direct access to a patient’s medical history via an electronic medical record, and the ability to remotely print discharge instructions. As digital health services are offered more frequently and as video interaction become easier to use, it will become so routine that it will become intuitive for both patients and physicians. This will result in increased use of digital health services, driving much-needed efficiency into the US healthcare system [2].

NewYork-Presbyterian Hospital is one of the nation’s largest and most comprehensive hospitals and is a leading provider of inpatient, ambulatory, and preventive care in all areas of medicine. With over 2600 beds and more than 6000 affiliated physicians and 20,000 employees, New York-Presbyterian receives more than 2 million visits annually, including more than 310,000 emergency department visits. In 2016, NewYork-Presbyterian, in conjunction with Weill Cornell Medicine and Columbia University Medical Center, launched a comprehensive enterprise-wide digital health program, which included the first mobile stroke unit on the East Coast and one of the first emergency department-based Telehealth Express Care Service in the nation [3, 4]. Both of these innovative programs have dramatically enhanced the way that one of the largest academic US healthcare systems provides emergency care to its patients.

## Case Presentations

### Digital stroke care

A person having a stroke loses approximately two million brain cells per minute when a blood blockage or rupture deprives the brain of oxygen [5]. Unfortunately, it is difficult to know without a CT scan whether the patient has a block or a bleed, and giving the wrong treatment can be fatal.

**Fig. 1:**
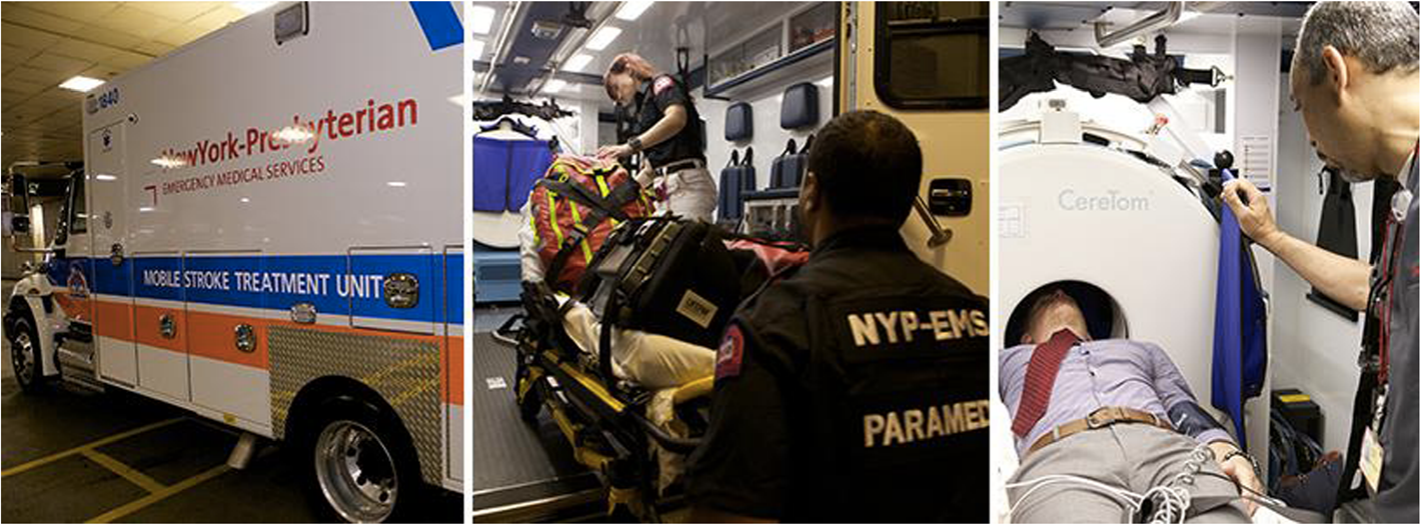
NYP Mobile Stroke Unit

Telestroke programs connect regional hospitals with a neurologist who can rapidly evaluate a CT image and consult with the physician on-site on the best course of action. Telestroke programs reduce the door-to-treatment time and have also improved patient outcomes [6, 7]. New York-Presbyterian took this care one step further by creating a Mobile Stroke Treatment Unit (MSTU). This specialized emergency vehicle, the first of its kind on the East Coast, has CT imaging capability onboard, is staffed by a neurologist, and is dispatched by the New York City 911 System via the FDNY directly to a patient showing signs of a stroke. The MSTU contains medications specific to diagnosing and treating strokes, allowing the team to deliver the right drug immediately upon diagnosis (Fig. 1).

Shortly, the cost of this rapid treatment will be reduced when the MSTU will wirelessly transmit the CT scan to a NewYork-Presbyterian neurologist who can cover multiple units while still seeing patients traditionally [2].

## Digital emergency care

**Fig. 2:**
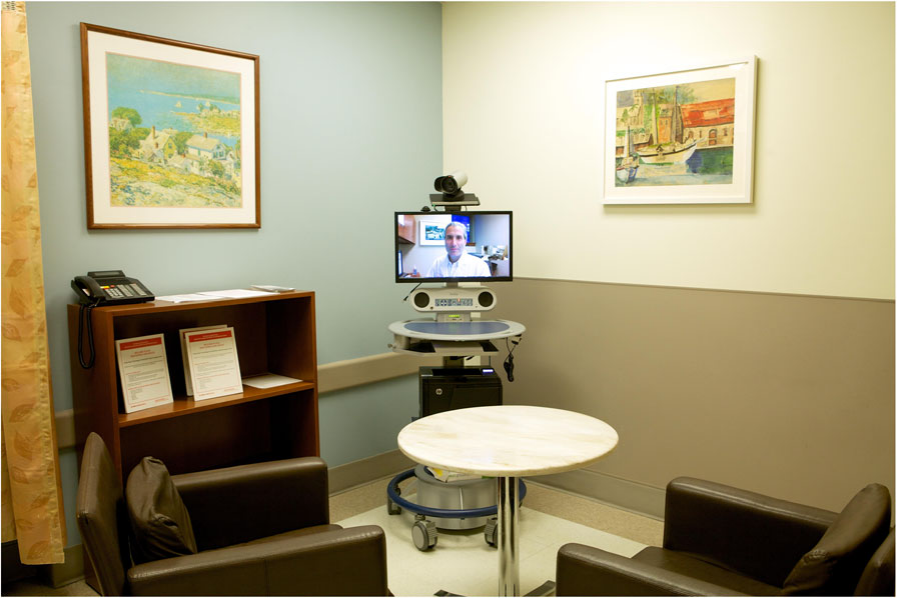
NYP ED Express Care Service Patient Room

Despite the passage of the Affordable Care Act (ACA), the number of emergency department visits has continued to rise nationally [8]. In the face of increasing volume and capacity challenges, healthcare systems must devise innovative solutions to ensure high quality and efficient care, while providing an outstanding patient experience. The innovative response of NewYork-Presbyterian and Weill Cornell Medicine was to create digital emergency department capability. One such example is the launch of the first Emergency Department-based Telehealth Express Care Service. Patients with minor complaints arriving at the emergency departments of New York-Presbyterian/Weill Cornell Medical Center and New York Presbyterian/Lower Manhattan Hospital are given the option of a virtual visit through real-time video interactions with a board certified Weill Cornell attending physician. The virtual visit is initiated after the patient has been triaged and had a medical screening exam. Telehealth Express Care Service complaints have included wound checks, upper respiratory infections, contusions, suture removals, and simple rashes. This virtual visit is conducted in a private room with a webcam/monitor, providing patients invaluable convenience and reducing the amount of waiting time in the emergency department (Fig. 2).

**Fig. 3:**
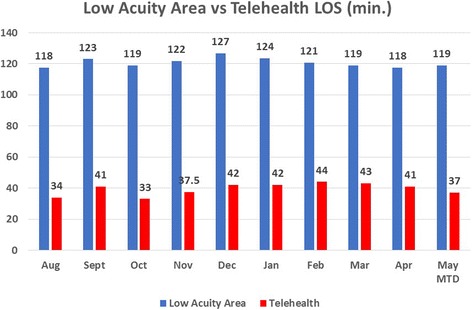
Length of Stay (LOS) for Low acuity area vs. Telehealth

The patient is then directly discharged from the private room with printed discharge instructions. There is no additional waiting period or checkout process. More importantly, the patients move through the entire Telehealth Express Care Service process in approximately 35–40 min from arrival to discharge. This compares to a typical 2 to 2.5 h visit for low acuity patients in the emergency department. The Telehealth Express Care Service has been embraced by our emergency department staff members. Implementation of the Telehealth Express Care Service has helped decant these patients from the typical emergency department workflow and allows emergency department staff members to focus on the higher acuity patients (Figs. 3).

We have also received great praise from patients. As of June 2017, approximately 2800 patients have benefited from this service and their patient experience scores have been in the 99th percentile. Each patient also receives a follow-up call from our nurses to ensure any questions they may have post discharge have been answered.

**Fig. 4:**
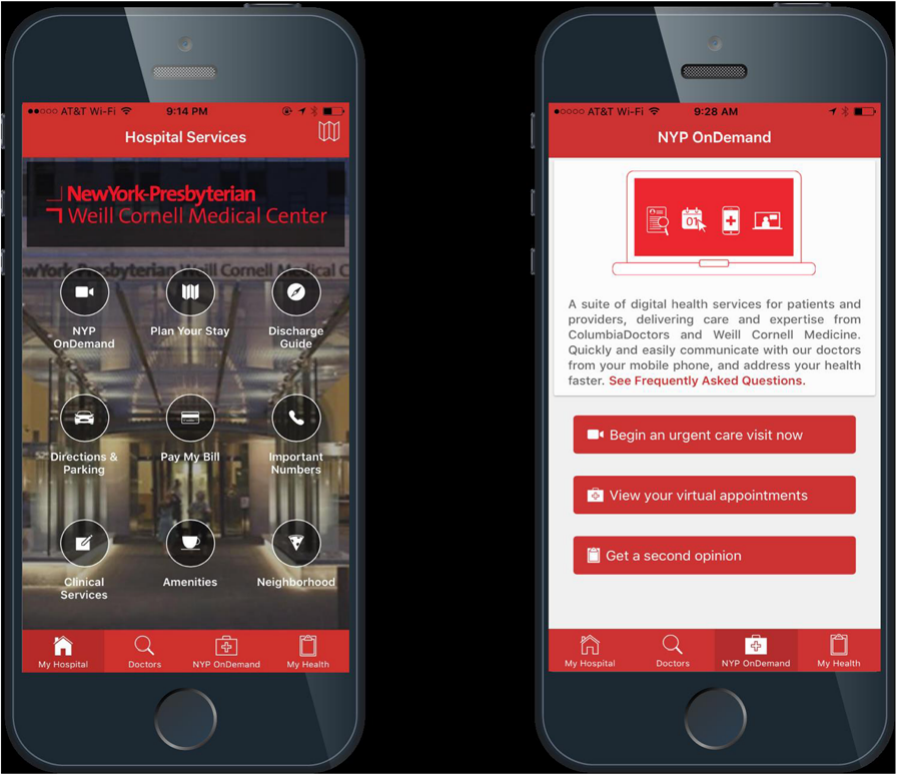
NYP On Demand and Virtual Urgent Care App

In an effort to provide care for patients with minor complaints at home, NYP launched the first On Demand Virtual Urgent Care App in New York City. This program enables patients to access our board-certified emergency physicians from the luxury of their home or workplace without having to make a trip to their doctor or urgent care center. Patients can see and speak with a board certified Weill Cornell Emergency Physician through a secure live video conference through their mobile device or home computer (Fig. 4).

As of June 2017, 70 patients have been seen via the NYP On Demand Urgent Care App. While this program is still in its early stages, we have received great feedback from both patient and provider participants. Patients who have used the service for emergency department visits through their phone have found it a preferable cost-effective and efficient process to making an in person physician emergency department visit.

## Lessons learned

While remaining innovative is crucial, it is equally imperative to maintain our standards and high quality of care. The technology used to provide these services is not novel or unique—it is the people, processes, and systems for patient outcomes that are the hallmark of a good digital health program. All the digital NYP Services discussed above have extensive quality control reviews to ensure excellence. In addition, each of these programs required comprehensive education of all staff members through several information sessions and regular updates. The commitment and adaptability of our staff has been integral to the success of these programs.

## Conclusions

The Telehealth Express Care Service and the Mobile Stoke Unit are just two emergency care programs in the comprehensive digital health portfolio offered by New York-Presbyterian Hospital. The appeal of digital health visits will inevitably increase as more services are offered remotely to be able to provide the right doctor to the right patient at the right time, locally, regionally, nationally, and eventually internationally. This comprehensive suite of services consists of digital second opinions in 80+ specialties, digital consults including but not limited to telestroke, telepsychiatry, telenursing homes, and telepediatrics, and finally digital video follow-up care. Primary care visits will become more data-rich as patients gain access to innovative tools like Tyto and MedWand, which allow patients to provide a remote physician with ear and throat images, breath sound, heart rate, blood pressure, and blood oxygen levels. NewYork-Presbyterian is also beginning to make pre-surgical visits with anesthesiologists and post-surgical checkups with physicians more convenient by scheduling these appointments as virtual video visits. In addition to being more efficient for the physician, they can turn a patient’s 4-h experience of traveling and waiting for a physician into a 10-min video appointment [2].

While telemedicine programs are in the early stages of implementation, we are confident that we will continue the expansion and build upon our initial successes. Innovation and technology have become invaluable in navigating the healthcare landscape. Telemedicine uniquely harnesses technology to provide patient access and convenient high quality care. We believe the expansion and enhancement of telemedicine programs will garner overall improvements in healthcare, from outcomes to provider satisfaction to patient experience.
